# Supplementary material for: Clinicopathological comparison of eccrine poroma and porocarcinoma: Ki-67 index is not a decisive factor
Source: PLoS One. 2025 May 30;20(5):e0324975. doi: 10.1371/journal.pone.0324975 (PMC12124553; doi:10.1371/journal.pone.0324975)
Supplement: Attachment — Submitted filename: Response to reviewers PLOS ONE.docx [file pone.0324975.s002.docx]

Dear Editor-in-Chief Boukerche,

Thank you for the valuable and helpful comments. We have now revised the manuscript PONE-D-24-44914 “Clinicopathological comparison of eccrine poroma and porocarcinoma: Ki-67 index is not a decisive factor” according to reviewers’ suggestions. We sincerely hope that our revised manuscript will be suitable for publication in the *PLOS ONE.*

Changes in the text are highlighted by Track change.

During the critical review process, Author noticed that one patient was errantly excluded from the statistical analysis. The patient’s diagnosis was labelled as basal cell carcinoma although it was eccrine poroma. This patient was added to the ”eccrine poroma” group in the analysis and new statistical tests were performed. In addition, it was verified that the information on local recurrences for eccrine poroma was confirmed only in two cases and for others no information was available. These changes are also highlighted in the revised manuscript.

Journal requirements:

1. We have revised the title page according to Journal’s style requirements.
2. Under the Finnish Biobank act (688/2012) the recipient of the material from Finnish Biobanks is not allowed to decide upon disclosure of the material in Open Access portals. The data are available at each Biobank with reasonable requests from the Biobanks.
   a. Helsinki Biobank: <https://helsinginbiopankki.fi/en/front-page> . Project code HBP2018003.
   b. Tampere Clinical Biobank. <https://www.pirha.fi/en/web/english/for-professionals/finnish-clinical-biobank-tampere> . Project code 2018-006
   c. Oulu Biobank Borealis: <https://oys.fi/biopankki/> . Project code BB_2019_3005

Reviewers’ comments to Author:

Reviewer 1:

The manuscript presents a study comparing clinicopathological factors between eccrine poroma (EP) and porocarcinoma (EPC), with a special focus on the Ki-67 index. The research is well-structured, addresses an important and rather underexplored topic, relevant statistical analysis has been applied.

1. The abstract provides a good overview but should also emphasize the clinical implications demonstrated by this study. Consider adding future research plans, if any are foreseen.

**RE:** The abstract has been modified according to suggestion.

2. The introduction is elaborate, scientifically well-supported by the relevant references. I would recommend adding more explanation the hypothesis here why Ki-67 was expected to differ between EP and EPC as it is somewhat elaborated only in the discussion section.

**RE:** We have now added explanation in the Introduction as advised.

3. Methods are reasonably described. However, the Ki-67 index analysis could be described even in a bit more detailed manner - for the audience not to be looking into the references 35 and 36. Taking into consideration the rarity of the disease, sample collection and potential biases in the process, sample size calculation / power analysis could also be explained to strengthen the study’s validity.

**RE:** We have now added the immunohistochemical staining in more detail in the Methods-section. As the disease under investigation is extremely rare, our study included all eligible cases available during the study period. Therefore, no prospective sample size calculation or formal power analysis was performed. We acknowledge this as limitation. Nonetheless, we believe the collected data provide meaningful insights into this rare condition and serve as a valuable contribution to the limited literature available.

4. Results and statistical findings are well-described. Consider discussing the high age in EPC patients as a possible confounding factor and if/how it may have influenced survival presented in Kaplan-Meier curves.

**RE:** This is now discussed in the Discussion more elaborately.

5. The discussion is interesting to read and reasonably supported by citing other studies. Consider emphasizing the clinical relevance of your findings, e.g., should Ki-67 be used in routine practice to prognose EPC?

**RE:** We have added a suggestion of the use of Ki-67 index in clinical practice in the Discussion.

Reviewer 2

1. Whole-exome sequencing identifies distinct genomic aberrations in eccrine porocarcinomas and poromas
To ensure the scientific rigor of the paper, please include the pathological diagnostic criteria for EP and EPC in the Methods section. How about including pathological images of all specimens stained with HE and Ki-67 as supplemental data?

**RE:** We have added a section describing the histopathological diagnosis of EP and EPC in the Methods-section. We are not allowed to include pathological images of all specimens as supplemental data due to the strict legislation of Finnish Biobanks.

Reviewer 3

1. As an academic editor, I would suggest it would be important to discuss these studies in the context of several studies such as the one below that shows distinct genomic aberrations in eccrine porocarcinomas and poromas including *TP53*, *NCOR1*, and *CDKN2A.*

Whole-exome sequencing identifies distinct genomic aberrations in eccrine porocarcinomas and poromas.  Puttonen M, Almusa H, Böhling T, Koljonen V, Sihto H. **Orphanet J Rare Dis.** 2025 Feb 13;20(1):70.

**RE:** We have included the suggestions in the Discussion
